# Effects of educational interventions based on the theory of planned behavior on oral cancer-related knowledge and tobacco smoking in adults: a cluster randomized controlled trial

**DOI:** 10.1186/s12885-024-11845-2

**Published:** 2024-01-08

**Authors:** Anoosheh Ghasemian, Katayoun Sargeran, Mohammad Reza Khami, Ahmad Reza Shamshiri

**Affiliations:** 1 Research Centre for Caries Prevention, Dentistry Research Institute, Tehran University of Medical Sciences, Tehran, Iran; 2 Department of Community Oral Health, School of Dentistry, Tehran University of Medical Sciences, Tehran, Iran

**Keywords:** Mouth cancer, Tobacco smoking, Prevention, Educational models, Theory of planned behavior

## Abstract

**Background:**

The theory of planned behavior (TPB) is an effective model for facilitating behavioral change. The aim of the present study was to evaluate the impact of TPB-based educational interventions on oral cancer-related knowledge and tobacco smoking behavior in an Iranian adult population in 2022.

**Methods:**

In this randomized controlled trial, a total of 400 healthy individuals were enrolled. The study was implemented in 20 urban health centers in the south of Tehran, Iran. The health centers were randomly allocated into two intervention groups. In group PowerPoint (PP), the participants received education through a 20-minute PowerPoint presentation complemented by a pamphlet. Group WhatsApp (WA) was educated via WhatsApp messages and images. Data was collected using a structured questionnaire at baseline, and at one- and three-month follow-ups. The outcomes were evaluated in terms of knowledge, tobacco smoking behavior, and the related model constructs i.e. intention, attitude, subjective norm, and perceived behavioral control. Generalized estimating equations (GEE) regression models were applied to assess the effect of interventions on repeated measurements of the outcomes. All analyses were conducted using STATA Software Version 17.

**Results:**

Out of all the participants, 249 (62%) were women. The mean and standard deviation (SD) of age were 39.67 and 13.80 years. Overall, group PP had a significantly higher score of knowledge compared to group WA (β = 0.43, *p* = 0.005). No significant differences were found between the groups with regard to tobacco smoking and the related TPB constructs, except for attitude with a higher score in group PP compared to group WA (β = 0.50, *p* = 0.004). At the three-month follow-up, both interventions had significant effects on increasing knowledge (β = 4.41), decreasing tobacco smoking (OR = 0.54), and increasing intention (β = 1.11), attitude (β = 1.22), subjective norm (β = 1.37), and perceived behavioral control (β = 1.08) (*P* < 0.001).

**Conclusions:**

Both interventions were effective in improving knowledge, tobacco smoking, and the TPB constructs after three months. Therefore, the application of both methods could be considered in the design and implementation of oral cancer prevention programs.

**Trial registration:**

The trial protocol was registered in the Iranian Registry of Clinical Trials (IRCT) on 04/03/2022 (registration number: IRCT20220221054086N1).

## Background

Oral cancer is a major global public health concern [1], ranking as the 16th most prevalent cancers worldwide [2]. According to the GLOBOCAN 2020 report, the incidence rate of lip and oral cavity cancers per 100,000 population was estimated 4.8 in the world and 1.4 in Iran [3]. The risk of developing oral cancer typically increases in individuals over the age of 40 years [1]. However, over the past decade, there has been a notable increase in the prevalence of oral cancer among young individuals and those under the age of 45 years [4].

Tobacco and alcohol consumption are the main risk factors that account for over 90% of oral cancer [5]. Tobacco consumption is one of the most important public health concerns worldwide [6]. In 2020, approximately 22% of the global population were reported to be tobacco users [6]. In Iran, the prevalence of tobacco, cigarette, and hookah smoking was 14%, 9.3%, and 4.5% in 2021, respectively [7]. Oral cancer predominantly affects individuals with lower socioeconomic status (SES) associated with some of the causal risk factors [8]. Considering that the majority of oral cancer cases are diagnosed at advanced stages with low survival rates, primary prevention strategies are crucial for decreasing the disease burden [9].

Providing public health education about oral cancer constitutes the most effective approach for the primary prevention of the disease [10]. Evidence suggests that educational interventions, such as one-on-one counseling sessions, distribution of leaflets and brochures, and the use of smartphone applications, can improve knowledge about oral cancer among both the general population and high-risk groups [11]. Face-to-face educational sessions utilizing PowerPoint presentations and printed materials as traditional methods of education, are often more instructive. On the other hand, smartphone applications offer a more interactive approach to learning, with the added benefits of message sharing and group discussions [12].

The major challenge in oral cancer prevention lies in the behavioral aspect of unhealthy habits, which can be addressed through the reinforcement of educational intervention methods [13]. The theory of planned behavior (TPB) is recognized as a comprehensive model for examining behavior [14] and considered an effective theory for behavioral change [15]. Educational interventions based on this theory can lead to sustainable behavioral modifications [15].

Knowledge serves as the foundational basis upon which the TPB concepts are constructed [16]. Behavioral intention, as the most important determinant of behavior, reflects the intensity of an individual’s willingness and effort to perform a certain behavior. Intention is influenced by three independent constructs. The first construct is attitude towards behavior, which refers to the positive or negative evaluation of performing a particular behavior. The second construct, subjective norm, represents the social pressure exerted by important individuals who either approve or disapprove the behavior, thereby influencing whether an individual chooses to perform or abstain from the behavior. The third construct is perceived behavioral control, which reflects the perceived ease or difficulty of performing a behavior, as well as an individual’s ability to execute the behavior according to potential obstacles [15, 16].

A number of epidemiological studies have investigated the impact of TPB-based educational interventions on improving knowledge about oral cancer or preventing cigarette, hookah, or tobacco smoking [17–22]. However, the majority of previous studies have been conducted on a small sample size [18, 19, 21, 22], within a narrow age group [17, 18, 20–22], or among high-risk populations [19, 21], without considering knowledge as the foundational basis of the TPB constructs [17, 18, 21, 22]. Existing studies have yielded inconsistent results regarding the effects of the interventions [17–22]. Furthermore, no research has been conducted to date that evaluates a TPB-based educational intervention on oral cancer-related knowledge and tobacco smoking among a broad age range in the general population, particularly in low SES groups who are at a higher risk of developing oral cancer [23].

Considering common risk factor approach [24, 25], the implementation of educational interventions and preventive strategies related to oral cancer, which have beneficial effects on general health and quality of life, can be integrated into the public health system [24]. These strategies can be also utilized in systemic disease prevention programs [24]. Given the aforementioned gaps in information, the aim of the present study was to evaluate the effectiveness of educational interventions based on the TPB on promoting oral cancer-related knowledge and preventing tobacco smoking in individuals aged 15 years and above from low socioeconomic background in Tehran, Iran.

## Methods

### Study design and participants

This parallel cluster, randomized controlled trial (RCT) with a 1:1 allocation ratio was conducted among the general population at urban health centers in the southern region of Tehran, Iran, in 2022. Regarding the inclusion criteria, participants were 15 years or older, resided in low-privileged or very low-privileged regions of Tehran, had no physical or mental diseases, a minimum literacy level of reading and writing, owned a smartphone with the social network application WhatsApp installed, desired to participate in the study and sign an informed consent form. The exclusion criteria were non-Iranian citizens, unwillingness to attend educational sessions, unwillingness to join social networks, and inability to use smartphone applications.

### Recruitment

**Fig. 1:**
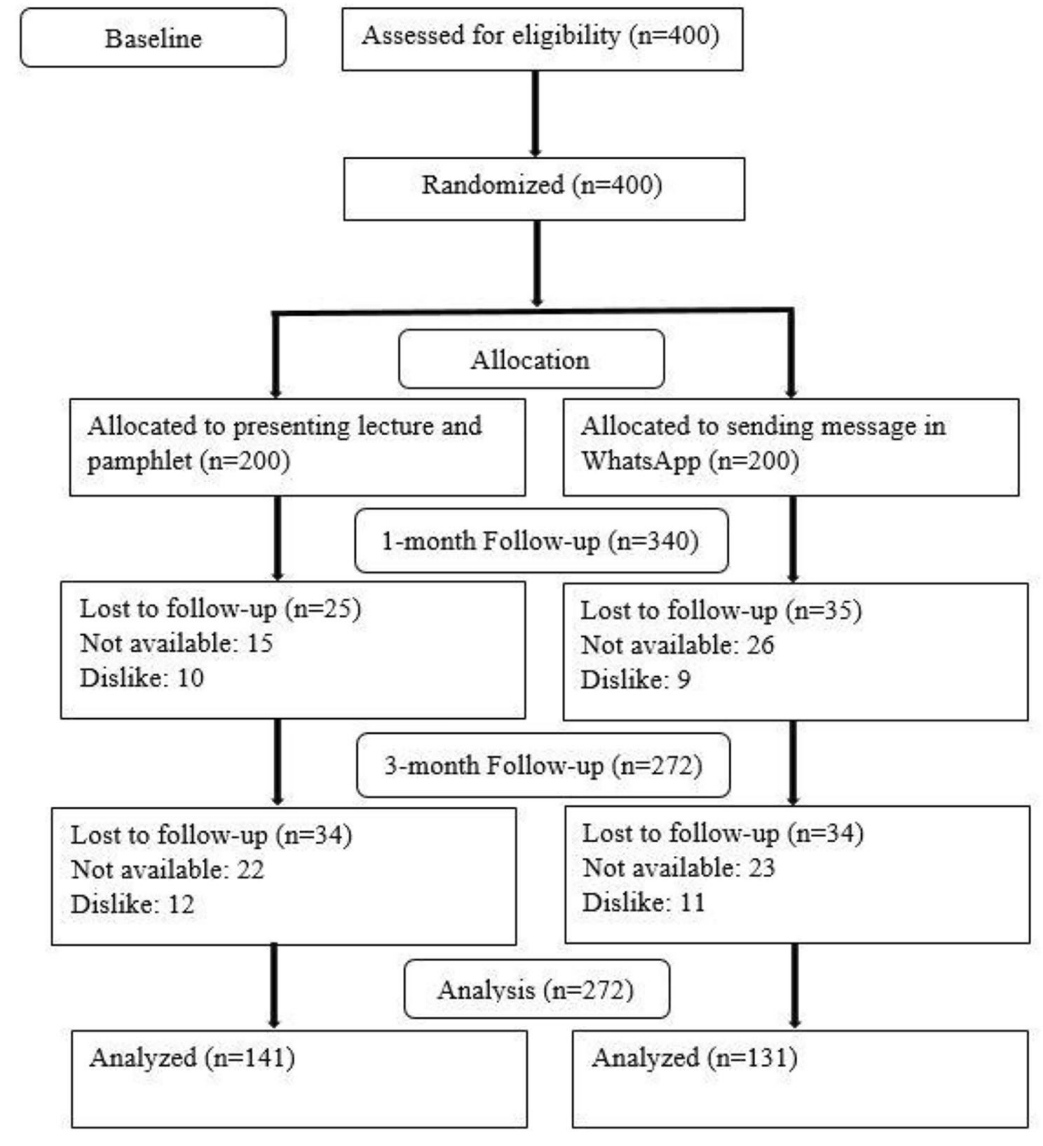
Flow chart of the trial

From 11/08/2022 to 25/11/2022, a total of 400 individuals were initially recruited. Follow-up assessments were conducted at one and three months post-intervention, with 340 and 272 individuals participating, respectively (Fig. 1).

### Sample size

A sample size of 400 was calculated using the formula for RCTs with an equivalence design. The following parameters were considered in this study: a type I error of 0.05 (z 1-alpha/2 = 1.96), power of 0.9 (z 1-beta = 1.28), delta of 1 (representing the difference and clinically acceptable limit), a standard deviation (SD) of 2 (representing the combined SD of both comparison groups) [22], a design effect of 2, and a dropout rate of 20%.

### Sampling and randomization

Sampling was carried out using the simple cluster random sampling method. Tehran is divided into five regions: privileged, relatively privileged, semi-privileged, low-privileged, and very low-privileged [26]. The low-privileged and very low-privileged regions were selected based on the research objective. Out of the 85 existing centers, 20 were randomly selected. The selected centers were randomly assigned into two intervention groups: PowerPoint (PP) and WhatsApp (WA), with 10 centers in each group. Assignment to the groups was performed through a simple randomization using Microsoft Office Excel software, which served as a random sequence generator. Each center and each group were assigned a unique code to maintain the concealment of the sequence. The codes were then entered into two separate columns, one for the centers and one for the groups. Subsequently, a colleague, who was blinded to the details, asked to execute the randomization command on the columns. Considering a sample size of 400, 20 participants were randomly selected from each health center’s list of individuals under their care. The selected individuals were then invited by health workers to participate in the study.

### Blinding

The data, identified by unique codes, was entered into the STATA software. This ensured that the data analyst remained unaware of the specific health centers and intervention groups associated with the data.

### TPB-based interventions

The participants in group PP were educated by a session of 20-minute lecture delivered via a PowerPoint presentation of 20 slides with educational messages and colored images. The participants in this group were invited to take part in one-session lecture on a specific day and time to ensure that everyone attends. They were also provided with one pamphlet for further reference. It was prepared by creating a brief format of the same messages and colored images of the PowerPoint slides. The participants were encouraged to review the pamphlet at home on a regular basis, specifically twice a week, over a three-month period.

The participants in group WA were educated by smartphones and the information was disseminated via messages and colored images in WhatsApp groups. A total of 20 JPG images (each containing three to four messages and images on average) were sent on a specific day at a specific time to ensure all participants had the opportunity to read them. The messages and images were prepared by converting the same messages and colored images of the PowerPoint slides into the JPG format to be able to send with WhatsApp. The participants were encouraged to review the materials at home on a regular basis, specifically twice a week, over a three-month period. 

The messages and images of both groups of PP and WA were pretested in a pilot study conducted over a two-week interval. This study involved 40 individuals, aged ≥ 15 years, who were randomly selected from two health centers (20 individuals in each center) that were not included in the main sample. Intervention for group PP was conducted in one center, and intervention for group WA in the other. The messages and images were delivered to the individuals for assessing potential executive problems or difficulties, as well as for practical methods to solve them, or for any modifications based on the results of the pilot study. 

In both group PP and WA, interactive group discussion and question-and-answer were held. For group PP, this took place at the end of the session, while for group WA, it occurred once a month. To further motivate the participants and encourage their continued participation, a dental care package containing a toothbrush and toothpaste was distributed among all individuals in both groups. Moreover, both interventions were reinforced through regular reminder phone calls (once every two weeks) to maintain contact with the participants and guide them regarding the regular use of educational materials. The participants in both groups had the opportunity to discuss any queries or ask any questions at the time of regular reminder phone calls.

### Educational content

The content of all the educational materials (PowerPoint, pamphlet, and WhatsApp) was designed based on the scientific literature and the TPB constructs. This was accomplished in two sections by an expert panel comprised of community dentistry specialists, as well as health education and promotion specialists. The first section (knowledge) emphasized on introduction to oral cancer, including definition, primary prevention, epidemiology, risk factors, signs and symptoms, and effects of risk factors on oral and general health considering the common risk factors [24, 25]. The second section (TPB constructs) emphasized on practical strategies and persuasive messages. The goal was to reinforce behavior and behavioral intention and to promote a positive attitude, subjective norm, and perceived behavioral control, targeted towards reducing or quitting tobacco smoking. 

In brief, attitude consisted of advantages of quitting tobacco smoking and disadvantages of not quitting tobacco smoking, as well as messages about believing that it is beneficial and good to quit tobacco smoking. Subjective norm consisted of the effects and roles of society, culture, family, and friends in confirmation and support of quitting tobacco smoking, as well as messages to family and friends for confirmation and support of quitting tobacco smoking. Perceived behavioral control consisted of methods to increase the ability to quit tobacco smoking and overcome existing obstacles, as well as messages about being easy and having the ability to quit tobacco smoking.

### Training of health workers

A group of 20 health workers, one from each health center, voluntarily participated in the study. The health workers were trained by a dentist using a PowerPoint presentation, equipping them with the necessary skills to effectively deliver the educational materials to the study population.

### Data collection

#### Instrument and outcome measurement

In the absence of a pre-existing standard version of a TPB-based questionnaire in the field of oral cancer, a structured interviewer-administered questionnaire was developed in accordance with a literature review and the concepts of the TPB questionnaire by Ajzen [27]. The questionnaire consisted of two parts. The first part contained questions about sociodemographic factors, including age, sex, educational level, occupation, household income, marital status, housing status, household size, and family history of cancer. The second part contained questions about knowledge and behavior, primary outcomes, and the TPB constructs, secondary outcomes, which are described below:

##### Knowledge

To evaluate knowledge, 11 questions were formulated, each pertaining to a different aspect of oral cancer prevention as covered in the educational content. Each correct answer was given a score of 1, while incorrect answers or responses of “I do not know” received a score of 0. Consequently, the possible scores ranged from 0 to 11.

##### Behavior

Two Yes/No questions were designed to assess cigarette and hookah smoking, with the following items: “I smoke cigarettes every day” and “I smoke hookah at least once a week”.

Three statements were designed to measure each of the TPB constructs, including:

##### Behavioral intention

“I intend to reduce or quit cigarette or hookah smoking within the next six months”; “I try to reduce or quit cigarette or hookah smoking”; and “I plan to reduce or quit cigarette or hookah smoking within the next month”.

##### Attitude toward behavior

“It is beneficial and valuable for me to reduce or quit cigarette or hookah smoking”; “It is pleasant and enjoyable for me to reduce or quit cigarette or hookah smoking”; and “It is good for me to reduce or quit cigarette or hookah smoking”.

##### Subjective norm

“Most people who are important to me and whose opinions I value (e.g., family, friends, dentist, and doctor) confirm that I should reduce or quit cigarette or hookah smoking”; “It is expected of me (by family, friends, and society) that I reduce or quit cigarette or hookah smoking”; and “I feel under social pressure to reduce or quit cigarette or hookah smoking”.

##### Perceived behavioral control

“It is possible for me and if I wanted I could reduce or quit cigarette or hookah smoking”; “I have complete control over reducing or quitting cigarette or hookah smoking”; and “It is mostly up to me whether or not I reduce or quit cigarette or hookah smoking”.

Responses to all statements were scored using a five-point Likert scale, which ranged from “1 = Completely disagree” to “5 = Completely agree”. The potential scores for each construct ranged from 3 to 15.

The structured questionnaire was consequently evaluated for validity and reliability. Face and content validity was assessed by eight professors of community dentistry, health education and promotion, and oral diseases. The content validity ratios (CVR) for knowledge, behavior, behavioral intention, attitude toward behavior, subjective norm, and perceived behavioral control were 0.89, 1, 1, 0.83, 0.83, and 0.83, respectively. The content validity index (CVI) for all measures was equal to 1. The reliability was evaluated in a pilot study conducted over a two-week interval. This study involved 40 individuals, aged ≥ 15 years, who were randomly selected from two health centers that were not part of the main sample. The Cronbach’s alpha coefficients for knowledge, behavioral intention, attitude toward behavior, subjective norm, and perceived behavioral control were 0.82, 0.84, 0.75, 0.80, and 0.77, respectively. Also, the intra-class correlation coefficients (ICC) for the measures were 0.95, 0.99, 1, 0.98, and 0.99, respectively.

### Ethical considerations

The study protocol was approved by the Research Ethics Committee of Tehran University of Medical Sciences (ethical code: IR.TUMS.DENTISTRY.REC.1400.189). Written informed consent forms were signed by the participants before recruitment in the study. For participants who were 15 to 18 years old, written informed consent forms were obtained from their parents and/or legal guardians. Individuals were assured that they could leave the study at any time and that their information would remain confidential.

### Statistical analysis

Categorical variables were described by number and percentage (%). The distribution of continuous variables was evaluated by graphical and statistical methods, and considered normal presenting by mean and SD. To investigate the effect of interventions on continuous and categorical variables, multiple linear and logistic generalized estimating equations (GEE) regression models with exchangeable structure of correlation were utilized, respectively. The models incorporated the group, time, and the interaction term. All models were adjusted for sociodemographic variables to eliminate residual confounding. The results were considered statistically significant at a *p*-Value < 0.05. All statistical analyses were performed in STATA Software Version 17.

## Results

Out of the total participants, 151 (37.75%) were men. Mean (SD) of age was 39.67 (13.80) years. Sociodemographic characteristics between the intervention groups were not significantly different, except household income (*p* < 0.001) (Table 1).

**Table 1:** Baseline characteristics by intervention groups among a sample of adults living in deprived areas of Tehran, Iran

	Group		*p-*Value
	PowerPoint	WhatsApp	
Age	39.89 ± 13.72	39.45 ± 13.90	0.75
Sex			
Male	80 (40%)	71 (35.50%)	0.35
Female	120 (60%)	129 (64.50%)	
Educational level			
Middle school and less	49 (24.50%)	44 (22%)	0.76
High school/Diploma	91 (45.50%)	98 (49%)	
Associate Degree and more	60 (30%)	58 (29%)	
Occupation			
Employee/Labor	30 (15%)	31 (15.50%)	0.91
Freelance/Self-employed	42 (21%)	35 (17.50%)	
Student	17 (8.50%)	20 (10%)	
Housewife	98 (49%)	102 (51%)	
Retired/Unemployed	13 (6.50%)	12 (6%)	
Household income			
Very low	38 (19%)	25 (12.50%)	**< 0.001**
Low	42 (21%)	75 (37.50%)	
Medium	66 (33%)	38 (19%)	
High	54 (27%)	62 (31%)	
Marital status			
Single/Widow	38 (19%)	37 (18.50%)	0.90
Married	162 (81%)	163 (81.50%)	
Housing status			
Personal	116 (58%)	113 (56.50%)	0.76
Rental	84 (42%)	87 (43.50%)	
Household size			
1–3	81 (40.50%)	88 (44%)	0.74
4	85 (42.50%)	82 (41%)	
5–7	34 (17%)	30 (15%)	
Family history of cancer			
Yes	54 (27%)	59 (29.50%)	0.58
No	146 (73%)	141 (70.50%)	

In both group PP and WA, there was a reduction in percentages of tobacco smoking, and an enhancement in mean scores of knowledge, intention, attitude, subjective norm, and perceived behavioral control during the period of the study (Table 2).

**Table 2:** Mean scores of knowledge, percentages of tobacco smoking, and mean scores of Theory of Planned Behavior (TPB) constructs at baseline, one-, and three-month follow-ups by intervention groups among a sample of adults living in deprived areas of Tehran, Iran

	Group					
	PowerPoint			WhatsApp		
	Baseline	First follow-up	Second follow-up	Baseline	First follow-up	Second follow-up
Knowledge	6.50 ± 2.18	10.19 ± 0.99	10.84 ± 0.42	6.13 ± 2.25	9.77 ± 1.24	10.69 ± 0.51
Tobacco smoking	49 (24.50%)	41 (23.43%)	26 (18.44%)	48 (24%)	40 (23.24%)	17 (12.98%)
Intention	2.66 ± 0.99	3.10 ± 1.08	3.56 ± 1.14	2.72 ± 0.96	3.19 ± 1.10	3.84 ± 1.15
Attitude	3.30 ± 0.99	3.86 ± 0.81	4.12 ± 0.80	2.94 ± 0.81	3.52 ± 0.77	4.25 ± 0.78
Subjective norm	2.77 ± 0.89	3.21 ± 0.88	3.78 ± 0.93	2.51 ± 0.95	3.11 ± 0.98	3.97 ± 1.15
Perceived behavioral control	3.19 ± 0.70	3.60 ± 0.71	3.93 ± 0.82	3.05 ± 0.79	3.58 ± 0.71	4.13 ± 0.86

Overall, knowledge score of participants in group PP was significantly higher than those in group WA (*p* = 0.005). Tobacco smoking and scores of intention, subjective norm, and perceived behavioral control did not significantly differ between the intervention groups. Overall, individuals in group PP had significantly higher attitude score than those in group WA (*p* = 0.004). All participants were observed to have significantly lower odds of smoking tobacco at the three-month follow-up compared to the baseline (*p* < 0.001). Scores of knowledge and all the TPB constructs significantly increased for all individuals at the one- and three-month follow-ups compared to the baseline (*p* < 0.001). (Table 3).

**Table 3:** The effects of intervention groups on knowledge, tobacco smoking, and the Theory of Planned Behavior (TPB) constructs over 3 months among a sample of adults living in deprived areas of Tehran, Iran

	Effect size*	95% CI	*p*-Value**
**Knowledge**			
Group PP (vs. Group WA)	0.43	0.13,0.72	**0.005**
Time (vs. Baseline)			
First follow-up	3.57	3.32,3.82	**< 0.001**
Second follow-up	4.41	4.14,4.68	**< 0.001**
**Tobacco smoking**			
Group PP (vs. Group WA)	0.86	0.49,1.51	0.60
Time (vs. Baseline)			
First follow-up	0.95	0.78,1.17	0.64
Second follow-up	0.54	0.43,0.69	**< 0.001**
**Intention**			
Group PP (vs. Group WA)	0.04	-0.34,0.42	0.84
Time (vs. Baseline)			
First follow-up	0.49	0.32,0.65	**< 0.001**
Second follow-up	1.11	0.91,1.31	**< 0.001**
**Attitude**			
Group PP (vs. Group WA)	0.50	0.16,0.84	**0.004**
Time (vs. Baseline)			
First follow-up	0.56	0.40,0.72	**< 0.001**
Second follow-up	1.22	1.03,1.41	**< 0.001**
**Subjective norm**			
Group PP (vs. Group WA)	0.26	-0.14,0.66	0.20
Time (vs. Baseline)			
First follow-up	0.51	0.27,0.74	**< 0.001**
Second follow-up	1.37	1.09,1.64	**< 0.001**
**Perceived behavioral control**			
Group PP (vs. Group WA)	0.19	-0.10,0.47	0.20
Time (vs. Baseline)			
First follow-up	0.56	0.42,0.69	**< 0.001**
Second follow-up	1.08	0.92,1.24	**< 0.001**

PP: PowerPoint, WA: WhatsApp

*regression coefficient (β) for knowledge, intention, attitude, subjective norm, and perceived behavioral control, and odds ratio (OR) for tobacco smoking

**p-Values were derived from generalized estimating equations (GEE) adjusted for age, sex, educational level, occupation, household income, marital status, housing status, household size, and family history of cancer

## Discussion

The present study was the first to investigate the effect of TPB-based educational interventions on improving oral cancer-related knowledge and tobacco smoking in an adult population with low SES, aged 15 years and above, in Tehran, Iran.

The results of the present study showed that participants who received education through lecture presentation and pamphlets demonstrated significantly higher knowledge compared to those who were educated via WhatsApp messages. A potential explanation might be attributed to the educational nature of in-person sessions [12]. In these settings, individuals have direct interaction with educators, which could foster a heightened level of focus and facilitate more effective learning. Moreover, pamphlet, as a complementary educational aid, may enhance the level of learning, as it is more readily available and can be more easily reviewed compared to messages sent by WhatsApp. Although smartphone applications have been recognized as more interactive methods [12], the non-use of such applications as a learning device make the participants less active to read messages carefully and communicate with others enthusiastically.

Consistent with our results, a number of national and international studies have confirmed the significant impact of educational interventions on improving knowledge about oral cancer [12, 19, 20, 28, 29]. However, our findings stand in contrast to an RCT conducted among Taiwanese with a history of betel quid chewing and smoking. In their study, no significant difference was noted between the intervention groups in terms of oral cancer-related knowledge [30]. The discrepancy in the results could be due to the high level of awareness among both groups within the high-risk population residing in remote areas of Taiwan. Generally, these areas have a higher incidence of oral cancer compared to Iran [3].

We found no significant differences between the intervention groups in terms of tobacco smoking and the TPB constructs, except for attitude. Our findings align with two RCTs conducted in the UK and China, where the interventions did not significantly influence cigarette smoking [17, 18]. The results of the present study revealed that there was a significant enhancement in knowledge among participants in both intervention groups. Additionally, there was a significant reduction in tobacco smoking and an increase in intention, attitude, subjective norm, and perceived behavioral control after a period of three months. The interventions were delivered by well-trained health workers who shared a similar socioeconomic and cultural background with the participants. The health workers provided consistent educational content. This approach was effective not only in enhancing knowledge, but also in promoting and encouraging behavioral change.

Furthermore, consistent with the results of many previous studies [19–22, 31–34], positive changes in knowledge, behavior, and the related constructs over time could be interpreted according to the theory concepts. The decrease in tobacco smoking could be attributed to the application of TPB, which is widely recognized as an effective model for facilitating sustainable behavioral change [15]. The presentation of the benefits associated with giving up tobacco smoking, along with the drawbacks of persistent tobacco smoking, led to an improvement in attitude towards cessation. Also, the elucidation of the roles played by society, culture, family, and friends in supporting the cessation of tobacco smoking, along with an explanation of the significance of social pressure, contributed to an increase in subjective norm. Moreover, the explanation of practical methods to boost self-efficacy and the ability to quit tobacco smoking, such as maintaining a regular personal program to foster self-control in quitting, led to an enhancement in perceived behavioral control.

However, contrary to our findings, some studies reported no significant improvement in oral cancer-related knowledge and tobacco smoking over time [17, 18, 35, 36]. The inconsistent results could be due to the varying durations of the studies, and the use of various educational materials or disparate behavioral models, each with differing efficacies for inducing behavioral change.

The present study benefits from a number of strengths. The first strength includes a large sample size drawn from the general public, encompassing a diverse range of age groups and both genders. Second, data was gathered using a valid and reliable questionnaire, which was structured in accordance with the conceptual and methodological considerations of a standardized TPB questionnaire. Third, the educational interventions were delivered in the form of both written and pictorial information, supplemented with discussions and reminders to enhance their appeal and effectiveness. The interventions were provided by well-trained health workers who shared the same background as the participants, thereby facilitating better comprehension. Another strength of this study was the application of the TPB, a model recognized for its effectiveness in examining and changing behavior, which could yield more precise and reliable estimations. Finally, given the clustered, correlated, and longitudinal nature of the data, the GEE models were utilized. This approach allowed for a thorough investigation of the reasonable effects of interventions on the repeated measurements of outcomes.

The present study had some limitations. The first limitation was that the study only included low-income adults who were serviced by health centers in the southern part of Tehran. This could potentially limit the generalizability of the findings. Another limitation of this study was the use of a self-report questionnaire, which could lead to inaccuracies in the responses due to personal, social, or ethical considerations. Third, the three-month follow-up period was relatively short, which might not be sufficient to evaluate behavioral changes.

## Conclusions

The findings of the present study demonstrated that knowledge related to oral cancer was significantly improved over time. Both interventions had a significant impact on reducing tobacco smoking and enhancing intention, attitude, subjective norm, and perceived behavioral control after a period of three months. The results of this study could be invaluable for oral health professionals in designing effective interventions that focus on a common risk factor approach. These interventions could be integrated into the existing public health system programs. Furthermore, the findings could provide policymakers with robust recommendations for formulating efficient policies and allocating financial support to facilitate the implementation of oral cancer prevention programs at both individual and population levels.

## Data Availability

The datasets used and analyzed during the current study are available from the corresponding author on reasonable request.

## Abbreviations

SES: socioeconomic status
TPB: theory of planned behavior
RCT: randomized controlled trial
SD: standard deviation
PP: PowerPoint
WA: WhatsApp
CVR: content validity ratio
CVI: content validity index
ICC: intra-class correlation coefficient
GEE: generalized estimating equations
